# How speech and language therapists and parents work together in the therapeutic process for children with speech sound disorder: A scoping review

**DOI:** 10.1111/1460-6984.13132

**Published:** 2024-11-18

**Authors:** Katherine Pritchard, Vesna Stojanovik, Jill Titterington, Emma Pagnamenta

**Affiliations:** ^1^ School of Psychology and Clinical Language Sciences University of Reading Reading UK; ^2^ The Speech Doctor UK

**Keywords:** home practice, parents, scoping review, speech sound disorder

## Abstract

**Background:**

Speech sound disorders (SSDs) are broadly defined as difficulty producing speech sounds in childhood. Reported prevalence of SSD varies from 2.3% to 24.6%, depending on how SSD is defined and the included age range. SSDs that do not resolve before age 8 can have a lasting impact on a child's academic achievements. The intensity of intervention for SSD is important to ensure effectiveness. However, there is a gap between the evidence base for intensity and speech and language therapists’ (SLTs) clinical practice. One way that SLTs try to bridge this gap is by working with parents. SLTs believe that working with parents/caregivers is vital for a child with SSD to make progress.

**Aims:**

To conduct a scoping review of the literature to provide a comprehensive picture of the perceptions, experiences and strategies underpinning collaborative working between SLTs and parents/caregivers of children (aged ≤ 5 years 11 months) with SSD to increase intervention intensity at home.

**Methods & Procedures:**

This scoping review was completed in accordance with PRISMA‐ScR guidelines. A systematic search of PubMed, PsycInfo, CINHAL, Web of Science, EBSCOhost and EThOS was conducted using synonyms of three key terms: SSD, Therapy, Parents. Key journals and papers were hand searched for unique papers. A total of 29 papers were included for review. Data were analysed using thematic synthesis to develop themes. These themes are discussed using the PAGER framework to identify advances, gaps, evidence for practice and areas for future research.

**Main contribution:**

Seven key themes were identified: individualization, setting expectations, daily life, parental knowledge, parental involvement, therapeutic relationships and supporting parents to deliver home practice. There has been an acceleration of research around working with parents of children with SSD, with increased consideration of effective adult coaching techniques. Parents value the parental and child relationship with the SLT and feel this supports the success of home practice. There is a need for further research, and guidance for SLTs working with parents of children with SSD to enable them to support parents to deliver home practice effectively.

**Conclusions & Implications:**

Emerging evidence supports the value of SLTs and parents working together to support home practice for children with SSD. The review highlighted the importance of SLTs allocating time to build positive therapeutic relationships with parents to support engagement in therapy. Approaching intervention, in particular, home practice, flexibly and in collaboration with parents, allows parents to fit home practice into their daily lives. Providing clear information to parents supports the fidelity of, and engagement in, home practice.

**WHAT THIS PAPER ADDS:**

**What is already known on the subject**

- There is a gap between what is recommended in the evidence base for intervention intensity for children with SSD and current clinical practice worldwide. SLTs try to bridge this gap through home practice and believe that working with parents/caregivers is vital for children with SSD to make progress. However, little is known about the best ways for SLTs to work with parents for this population.

**What this paper adds to the existing knowledge**

- Training parents to be implementers of intervention in a personalized and flexible way is important and valued by parents and SLTs. Parents value understanding the clinical rationale behind the intervention approach and benefit from explicit instructions for home practice, including discussion, written information, observation and feedback. Therapeutic relationships take time to develop and impact parental engagement in home practice.

**What are the potential or actual clinical implications of this work?**

- The findings of this study highlight existing knowledge which will support SLTs to work optimally with parents to implement home practice for their child with SSD. It highlights the importance of taking time to foster working relationships with parents to support effective home practice. The review identifies gaps in the current skills and knowledge of SLTs, highlighting the need for further research, support and guidance for SLTs in their work with parents, as well as implications for the development of the SLT pre‐registration curriculum.

## INTRODUCTION

Children with speech sound disorders (SSDs) can have difficulties with any combination of:

perception, articulation/motor production, and/or phonological representation of speech segments (consonants and vowels), phonotactics (syllable and word shapes), and prosody (lexical and grammatical tones, rhythm, stress, and intonation) that may impact speech intelligibility and acceptability. (McLeod et al., 2013: 1)

The reported prevalence of SSD in childhood varies from 2.3% to 24.6% (Beitchman et al., 1986; Eadie et al., 2015; Jessup et al., 2008; Law et al., 2000; Shriberg et al., 1999). This wide variation is due to differences in studies on how SSD was defined, which age was considered and which assessments were used to diagnose SSD. Broomfield and Dodd (2004) found that ‘speech difficulties’ were the most common area of difficulty amongst referrals to community paediatric speech and language therapy (SLT) clinics, with the majority of referrals for children aged 2–6 years. A survey of SLTs in the UK found that almost half of the SLTs reported more than 40% of their caseload as having ‘phonology problems’ (Joffe & Pring, 2008: 159). Another survey in South Africa found that SLTs reported between 40% and 60% of their caseloads were children with SSD (Pascoe et al., 2010).

Children with SSD at school age are more likely to have difficulties learning to read and write (Wren et al., 2021). SSD may also ‘be associated with limitations and restrictions’ across at least six of the nine areas of activity and participation of the International Classification of Functioning, Disability and Health (McCormack et al., 2009: 163). Wren et al. found that SSD persisting past the age of 8 can have a lasting negative impact on a child's academic achievements (Wren et al., 2021) and peer relationships (Wren et al., 2023). Children with SSD past age 8 are also twice as likely to report self‐harm with suicidal intent than those without SSD at age 8 (McAllister et al., 2023). This highlights the importance for intervention for SSD to be prioritized and delivered with fidelity to the evidence base, in a timely manner. There is currently a gap between evidence‐based practice recommendations for intensity of intervention for SSD and SLT practice in the UK and other Western countries (Hegarty et al., 2021; Joffe & Pring, 2008; Sugden et al., 2018b). This is especially important in relation to the recommended intervention intensity. Intervention intensity is calculated using dose (the number of teaching episodes per session) × dose frequency (i.e., how many sessions within a specified time) × total intervention duration (time over which intervention is provided) (Warren et al., 2007). The average dose frequency recommended for children with phonological SSD is 2–3 sessions per week (Sugden et al., 2018b) and for children with Childhood Apraxia of Speech this is even higher, with up to four sessions per week recommended (McCabe et al., 2020). This frequency is difficult to achieve in a typical service model in the UK where the number of sessions available for children are restricted (McFaul et al., 2022; RCSLT, n.d.; RCSLT & ICAN, 2018). One way that SLTs may try to bridge this gap is by working collaboratively with parents to increase the intensity of intervention through home practice (Hegarty et al., 2021; Joffe & Pring, 2008). SLTs believe that working with parents is vital for a child with SSD to make progress (Furlong et al., 2018; Oliveira et al., 2015; Pappas et al., 2008; Sugden et al., 2018a) yet there is little understanding about the best ways to do so (Sugden et al., 2018a).

Therefore, a comprehensive review of the existing evidence base is needed to address how SLTs currently support parents in their role as implementors of intervention, as well as determining what is known about how parents and therapists experience and perceive this role. It is important for SLTs to understand what techniques and strategies are available in the existing evidence to support them in their work with parents.

## AIMS AND OBJECTIVES

The aim of this scoping review is to identify and synthesize the existing literature to provide a comprehensive picture of the perceptions, experiences and strategies underpinning effective collaborative working between SLTs and parents of children, aged ≤ 5 years 11 months (5;11) with SSD. The key focus is on how parents are supported to develop their role as implementers of intervention. The following questions acted as reference points for our discussions throughout the review:

- How do SLTs support parents to develop their role as implementers of intervention in clinical sessions alongside the SLT, and in home‐based activities, for children diagnosed with SSD up to age 5;11?
- How do SLTs work with parents to ensure that the approach and intensity of intervention are delivered with fidelity in the home environment?
- How do SLTs experience and perceive working with parents?
- How do parents experience and perceive working with SLTs?

## METHOD

A scoping review methodology was chosen to identify the range, scope and types of available evidence within the existing literature (Munn et al., 2018). The selection of the methodology was guided by Arksey and O'Malley (2005), the Joanna Briggs Institute (JBI) (Peters et al., 2020) and a PRISMA extension for scoping reviews (PRISMA‐ScR) methodologies (Tricco et al., 2018). It was also anticipated that the review would be useful in informing clinical practice and in informing the development of university curricula for effective training of SLTs (Arksey & O'Malley, 2005; Bradbury‐Jones et al., 2022; Levac et al., 2010). This is important to enable SLTs to understand the complexity of their interventions for children with SSD (Noyes et al., 2023). Moreover, a scoping review can identify gaps in the research which could then inform ongoing research in the area (Bradbury‐Jones et al., 2022; Tricco et al., 2018).

As recommended, a team approach was taken with the research team meeting regularly (fortnightly) to discuss each stage of the review (Pollock et al., 2023).

The study was registered on the Open Science Framework (OSF) in February 2023 (Pritchard et al., 2023) (https://doi.org/10.17605/OSF.IO/B7WZH)

### Search and inclusion/exclusion criteria

The search strategy was proposed initially by the first author and then reviewed and adapted in consultation with the research team during December 2022 and January 2023. Search terms, inclusion and exclusion criteria were guided by the PRISMA‐ScR headings of population, concept and context (PCC) (Table 1; and see Appendix 1 in the Supporting Information section) (Tricco et al., 2018) and fell under three headings: SSD, therapy and parents. Multiple diagnostic labels fall under the umbrella term SSD and historically many different terms have been used to mean different things. Therefore, it was appropriate to use a wide range of terms for SSD (as agreed by the research team with reference to the literature) to capture as much of the existing literature as possible within the database searches. Review papers were also consulted to ensure relevant alternative terminology for parents/carers (Melvin et al., 2020; Tosh et al., 2017). The search terms were further checked for relevance and inclusion of key terms after running searches on PsycInfo and PubMed databases, as recommended within the JBI guidance (Peters et al., 2020) (for a full list of search terms, see Appendix 1 online). A subject specific librarian was also consulted throughout the process to support with database searching and when refining the search criteria.

**TABLE 1:** inclusion/exclusion criteria following the population, concept and context (PCC) framework.

**PCC**	**Inclusion criteria**	**Exclusion criteria**
Population/participants	Either: Parents of children aged up to 5;11 with a diagnosis of SSD OR SLTs working with children with SSD aged up to 5;11and their parents	Parents and SLTs where all of the children and young people over age 5;11 Parents of children with a diagnosis of any genetic or sensory disorder Parents of children with a cognitive impairment SLTs who do not work with children with SSD
Concept	Studies must include direct therapy delivered to the child by a SLT Must involve working with parents alongside direct therapy in some way And either Reports parent or SLTs perceptions and experiences of working together (research questions 3 and 4) OR Details any methodology of working with parents within the therapy session or to follow‐up afterwards (research questions 1 and 2)	Studies that do not involve direct therapy with an SLT/SLT student Studies that do not involve working with parents and/or providing homework in the session
Context	Services where SLTs and parents work together within the session Studies published up to and including February 2023 Studies published in English	Adult speech and language therapy services The person who is likely to be the main intervenor outside the therapy is not the parent

**^Source:^ :**Tricco et al. (2018).

As well as database searches, two theses repositories (EthOS and Enterprise) and key journals (American Speech–Language–Hearing Association journals, *Child Language Teaching and Therapy*, *International Journal of Language & Communication Disorders*) were searched using the search term headings. The reference lists of key evidence synthesis papers were hand searched for any articles that had not been found in the other searches (Melvin et al., 2020; Sugden et al., 2016; Tosh et al., 2017). The final searches were completed on 10 February 2023 (for full details of the search strategy, see Appendix 1 in the Supporting Information section).

As per scoping review methodology, the quality of the included papers was not appraised and a variety of sources was accepted, including book chapters, theses and evidence synthesis papers (Bradbury‐Jones et al., 2022; Peters et al., 2020). Due to lack of resources, the scope of the project and limited linguistic skills of the research team, it was not feasible for studies to be translated into English from other languages; hence only studies published or translated into English were included. No other limits were added to the searches to ensure that the search captured the full breadth of the existing literature, including how the field may have changed over time as recommended in the PAGER framework (Bradbury‐Jones et al., 2022). Where results were unable to be sourced by the researchers, they were sourced, where possible, via inter‐library loans, and in the case of one thesis through direct contact with the author (Bear, 2022).

### Selection of included evidence

**FIGURE 1:**
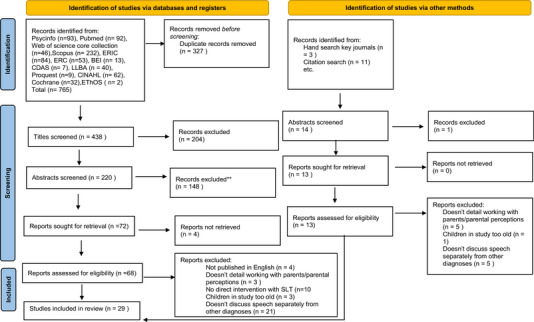
PRISMA‐ScR flow diagram. Source: Page et al. (2021).

Following the searches, 779 sources were identified. Search results were exported to EndNote. A total of 341 duplicates were removed using the tool on EndNote and through manual searching. The remaining 438 were screened at the level of the title using the inclusion/exclusion criteria, by author KP and then all 438 were cross checked by author EP. A total of 234 abstracts were then screened by author KP with the rest of the research team reviewing a third of the abstracts each. A total of 81 full text sources were then reviewed by author KP and divided between the rest of the research team for second reviewing. The sources were reviewed independently at each stage and then compared. Any discrepancies were discussed between author KP and the second reviewer and a consensus for inclusion/exclusion of each study was agreed. If a consensus could not be reached by two reviewers another member of the research team was consulted until a decision was made. In the case of any unresolved disagreements the source was taken through to the next stage. Sources were excluded due to them not being published in English, not detailing working with parents/parent's perceptions, no direct SLT intervention reported, children in the study being too old or not discussing SSD as a discrete diagnosis. A total of 29 sources were included in the data extraction phase (for the PRISMA flowchart, see Figure 1).

### Data extraction

Key information and relevant data were extracted from all 29 sources using an adapted version of Rodgers et al. (2022) scoping review data extraction form. Following a pilot of the form with author KP and author EP, it was agreed that due to the different methodologies included within the sources, it was appropriate to have two different data extraction forms, depending on the methodology of the study, one for intervention studies and another for surveys/qualitative studies. Instructions for extraction were written by author KP to ensure a consistent approach (Pollock et al., 2023) (for forms and instructions, see Appendix 2 in the Supporting Information section). For evidence synthesis papers (e.g., Sugden et al., 2016), the data were extracted from the results and the discussion sections only as these were considered the novel piece of research, rather than extracting any original data from individual studies included in the synthesis. This ensured that one piece of data was not considered twice, as there was some overlap between included studies. As recommended by Pollock et al. (2023) a second member of the research team (Author EP) extracted data independently from six of the 29 sources (Pollock et al., 2023). Papers chosen for double extraction were from a variety of methodologies. Data extraction was then compared. This process ensured consistency and verified the approach. It was found that whilst the data extracted were comparable, some data extracts were mapped to different research questions by the two reviewers. This did not impact on data analysis, as an inductive approach was taken for initial coding across the complete dataset (see below). Completed data extraction forms are available in the Supporting Information section.

### Analysis

Analysis focused on developing key themes and subthemes. The data were analysed using thematic synthesis, an appropriate method for the synthesis of heterogenous data from different methodologies (Thomas & Harden, 2008). It was chosen above other potential analysis approaches, such as framework analysis (Gale et al., 2013) and qualitative content analysis (Graneheim & Lundman, 2004) because it goes beyond description and allows the researchers to consider how the synthesized findings can be used to make recommendations for practice (Thomas & Harden, 2008).

The analysis followed a four‐stage approach:

1. Line by line free‐coding of all the extracted data, to capture the meaning and content.
2. Checking the codes for consistency and assigning new codes as relevant.
3. Developing descriptive themes by grouping codes.
4. Developing analytical themes (Thomas & Harden, 2008).

Stages 1–3 were completed by KP. To support the validity of stage 3, a summary of the descriptive themes and the reasoning behind these was written and shared with the rest of the research team and a final version was agreed. At this point the themes interpreted the data but were not mapped to the research questions. Consequently, the development of the final analytical themes involved independent synthesis of these descriptive themes by all of the research team who used the research questions as a reference. To ensure consistency of approach, KP developed guidelines to support this process (see Appendix 3 in the Supporting Information section). Table 2 shows an example of how data were given a code which then linked to the themes. The analytical themes from all authors were then collated and summarized by KP and final themes were agreed by the team through discussion.

**TABLE 2:** Example of mapping data from codes to analytical themes, from Lefebvre et al. (2017).

Data	Free code	Relevant descriptive theme	Relevant analytical theme
Although a general sound pattern was targeted each week, clinicians used the coaching time to individualize this to each child/family. (p. 63)	Approach adapts to the child/family	Flexibility	Individualization

These themes (patterns/P) were then considered in relation to the PAGER framework to consider advances in practice (A), gaps in the evidence (G), evidence for practice (E) and recommendations for future research (R) (Bradbury‐Jones et al., 2022).

### Author reflexivity

To enhance the rigour of the approach we reflected on our personal responses throughout the process. KP, EP and JT are all practicing SLTs who have experience of working with parents. EP, JT and VS have worked on/are working on other projects that examine parent‐mediated interventions with SSD and other clinical groups. The view across the team is that parent involvement can be effective and support the delivery of good outcomes from an intervention, but also, that this is not always the case. The team were open to all findings to better understand how SLTs work with parents and how both parents and SLTs view this process.

## RESULTS

### Description of the studies

Of the included sources, 13 were from Australia, eight from the United States, three from the UK, three from Canada, one from Germany and one from Thailand. Gathering sources from around the world and from different contexts allowed the review to be informed by a diversity of services and experiences, including studies conducted in regions with more experience of working effectively with parents than in most of the UK, such as studies coming from rural parts of larger countries. The date of publication ranged from 1989 to 2022 with just over half published after 2010. Most of the sources included intervention approaches, either intervention studies (*n* = 13) or narrative descriptions of intervention approaches (*n* = 4). Of these, two were mixed methods papers looking at an intervention and the parental experiences of this. Five papers were surveys of SLTs, four were semi‐structured interviews (three with parents and one with SLTs) and one was a systematic review (for full characteristics of the included studies, see Tables 3a, 3b, 3c).

**TABLE 3a:** Characteristics of the included studies: Intervention studies.

**First author and year**	**Study aim**	**Study design and method**	**SSD diagnosis of children**	**Nature of intervention**	**No. of participants**	**Context (language, age of children, therapy setting)**	**Country**
Bear, 2022	To compare how SLTs and parents support children with SSD and analyse what parents and SLTs discuss in intervention sessions	Longitudinal observation study	Moderate to severe SSD (based on PCC)	Eclectic approach (including phonetic and phonological approaches)	13 parent–child dyads	At least one parent fluent in English 3;0−6;11 Clinic: 4–9 sessions of therapy (mean = 5), one follow‐up session, 20 weeks post‐intervention	UK
Bowen, 1998	To provide a detailed case description of typical response to PACT approach	Single case study	Moderate developmental phonological disorder and a severity rating of 3.75	Broad‐based phonological eclectic approach	One child + mother	Monolingual, Australian English speaking 4;4 at initial assessment Sessions delivered in clinic + homework, 27 sessions in 10‐week blocks with breaks over 17 months	Australia
Bowen, 1999	To provide a narrative description of PACT intervention approach	Narrative review of non‐randomized group comparison study	Phonological disability. No HI, language impairments or other SLCN	Broad‐based phonological eclectic approach	14 treated children + parents, eight untreated	Monolingual, Australian English speaking 4;4 at initial assessment Sessions delivered in clinic + homework, 27 sessions in 10‐week blocks with breaks over 17 months	Australia
Bowen, 2004	To review the family education and homework aspects of PACT	Narrative review of PACT approach	Phonological disability. No HI or language impairments or other SLCN	Broad‐based phonological eclectic approach	14 treated children + parents, eight untreated	Monolingual, Australian English speaking 4;4 at initial assessment Sessions delivered in clinic + homework, 27 sessions in 10‐week blocks with breaks over 17 months	Australia
Broen, 1990	To determine if children's phonological skills improve if taught by parents versus no treatment and if children's skills improve if taught by parents versus not participating in the programme at all	Non‐randomized group comparison study	Delayed phonological development	Phonological treatment programme with parent–child classes	12 in experimental group, eight in comparison groups	Language/s not specified 43–60 months Clinical setting	USA
Eiserman, 1990	To compare costs and effects of home‐based versus clinic‐based intervention programme	RCT	Moderate speech disorders	Mix of phonetic and phonological approaches + language training and training in other areas where development was behind	40 children	Monolingual (language not explicitly mentioned) 3–5‐year‐olds Intervention at home	USA
Eiserman, 1992	2‐year follow‐up of 12 children from Eiserman, 1990	Second year follow‐up to 1990 RCT study	Moderate speech disorders	Mix of phonetic and phonological approaches + language training and training in other areas where development was behind	12 children	Monolingual (language not explicitly mentioned) 3–5‐year‐olds Intervention at home	USA
Flanagan, 2018	To determine the effectiveness of variations of core vocabulary therapy	Case series design comparing pre‐ and post‐intervention data	Inconsistent phonological disorder	Core vocabulary therapy	Five children	Monolingual, Australian English Under 6 years Clinic with homework over 7–12 weeks	Australia
Günther, 2010	To investigate if incorporating contingency management into articulation therapy increases home practice	Non‐randomized group comparison study	Articulation disorder	Articulation therapy ± contingency management	91 children	German‐speaking children 4–6 years Delivered in preschools and clinics by student SLTs, eight sessions for 45 min over 4 weeks	Germany
Hartmann, 1989	To describe a speech and language therapy home programme for parents to use with children with SSD	Description of approach for parents and paraprofessional (intervention manual)	For children with a range of SSDs	Home based articulation programme	n.a.	For preschool and school‐age children	USA
Lefebvre, 2017	To describe the Wee Words approach and the analysis of pre–post‐treatment measures in relation to speech and expressive vocabulary	One group comparing pre‐ and post‐intervention data	Suspected Childhood Apraxia of Speech (CAS)	Parent–child group working on motor‐based principles and sensory strategies	38 attended; data from 32 children included	Exposure to English at home (but does not need to be 1st lang) 24–40 months 6 × 60‐min therapy sessions and two parent education sessions	Canada
Lancaster, 2010	To test an eclectic approach to therapy conducted under conditions similar to real clinical practice	RCT	Phonological delay/disorder	Eclectic approach as part of regular clinical practice	27 children across the two studies	English is first or only language 3;4–5;10 15 30‐min sessions in one condition and parent training in the other	UK
Lim, 2020	To look at the effectiveness of a parent training programme for children in remote areas	Mixed methods: single case experimental design followed by parental interviews	CAS	Dynamic temporal and tactile cueing	Four parent–child dyads	English as a first language Three of the children were under 6 4 × weekly 1–1.5‐h parent training sessions, 4 weeks direct treatment, 2 weeks break, 4 weeks treatment and 4 weeks maintenance	Canada
Pumnum, 2015	To determine the effectiveness of a community based SLT model in reducing articulation errors	One group pre–post‐test design	SSD of known origin (following repaired cleft lip and palate)	Articulation therapy	Six children	Language not specified 5–8 years old (one child included was under 6 years) 3‐year project including speech camps at hospital and home visits	Thailand
Ruscello, 1993	To compare a computer intervention programme delivered by parents and SLTs	RCT	Children included scored < 15th percentile on Khan–Lewis phonological analysis	Minimal pair therapy via computer programme	12 children	Language not specified Age 4;1–5;8 (mean age = 5) Total of 16 h delivered as twice weekly 1‐h sessions, group 1 = 2 with SLT, group 2 = 1 with SLT, 1 with parent	USA
Rvachew, 2015	To determine the effectiveness of a home programme focused on vocabulary development versus an approach focused on speech production in achieving gains in phonological awareness and speech production accuracy	RCT	Developmental phonological disorder	Two interventions: one targeting articulation and one on perception/phonological awareness and vocab knowledge	72 children recruited, 65 attended	French‐speaking children 4−5‐year‐olds Intervention received over 12 weeks	Canada
Scherer, 2008	To determine whether a parent can be trained on early intervention programme and if this results in positive change in speech	Non‐randomized group comparison study	SSD of known origin (unilateral cleft)	Parent implemented focused stimulation	20 children (10 with cleft and 10 typically developing controls) and their mothers	Language not specified 14–25 months 2–4 45‐min sessions	USA
Sugden, 2020	To determine whether a parent can deliver multiple oppositions therapy competently and with confidence	Single case series with multiple baselines replicated across five participants	Moderate to severe phonological impairment	Multiple oppositions	Five parent–child dyads	English as strongest, or equally strongest, language Children aged 3;0–5;11 One 60 min clinic session a week for 8 weeks	Australia
Watts‐Pappas, 2010	To describe a family‐friendly intervention approach	Narrative description of approach, including a single case study	Moderate–severe inconsistent phonological disorder (in case study)	Family‐friendly approach to use alongside intervention approaches (case study uses core vocabulary)	One child	Language not specified 3 years 7 months Clinic based	USA

*Note*: CAS, childhood apraxia of speech; HI, hearing impairment; PACT, parent and children together; PCC, percentage consonants correct; RCT, randomized control trial; SLCN, speech, language and communication needs; SLT/SLP, speech and language therapist; SSD, speech sound disorder.

**TABLE 3b:** Characteristics of included studies: surveys and qualitative study designs.

First author and year	Study aim	Study design and method	SLTs/parents	No. of participants	Further participant details	Country
Furlong, 2018	To explore clinical decisions made by SLTs working with children with SSD	Semi‐structured in‐depth interview study: analysed using thematic analysis	SLTs	11	Working with more than one child with SSD. 10 women, one man. 20–70% of participants caseloads were SSD. SLTs worked across private, public and university services	Australia
Joffe, 2008	To gather evidence about clinical practice in the UK for children with speech and language problems	Survey	SLTs	98	Working in preschool and primary aged children in locations across the UK. Seven participants specialized in phonological delay/disorder	UK
McAllister, 2011	To examine parents’ expectations and experiences accessing and participating in SLT for the child	Semi‐structured interviews, analysed using the phenomenological approach^a^	Parents	13 parents	Child's mean age = 55 months, 72 boys, 37 girls, mostly mothers (94.5%), mostly monolingual with less than 10% multilingual	Australia
McLeod, 2014	To describe current practices of SLTs working with children with SSD in Australia	Survey	SLTs	231 (218 valid responses)	Invited whilst at a conference 71.7% response rate. 98.7% female, 45.9% parents, from across Australia. 54.1% SLTs worked fulltime and 45.9% worked part‐time	Australia
Pappas, 2018	To determine to what extent parents are involved with SLT for children with SSD as well as the SLTs attitudes, experiences and if there are differences between beliefs and practice	Survey	SLTs	277	12.6% response rate. 97% female, 37% parents, working across health, private and education settings. Half had majority SSD on caseload. Between 1 and 39 years of experience as an SLT	Australia
Sugden, 2018	To describe how SLTs involve parents in intervention for phonology‐based SSD and the motivation behind this	Survey	SLTs	288	99.3% female, 98.7% usually involved parents in intervention	Australia
Sugden, 2019	To examine how parents of children with SSD experience home practice with their child	Semi‐structured interviews, analysed using qualitative content analysis	Parents	Six mothers	Children aged 3–6 years, have experienced home practice alongside clinic‐based service	Australia
Tambyraja, 2020	To examine how SLTs follow‐up about home practice, what do SLTs think are successful strategies, what factors impact whether an SLT follows up	Survey	SLTs	156	SLTs had at least one child with SSD on their caseload, 60% always provide homework	USA
Watts‐Pappas, 2016	Examining how parents are involved with their child's intervention at home and their beliefs and experiences	Multiple‐sequential interviews (three interviews in total for each participant), analysed using thematic networks analysis and framework analysis	Parents	Seven	Six mothers, one father, recruited by SLTs, child ages 3;0–5;1 with mild–moderate SSD. Conversational English	Australia

*Notes*: ^a^This paper also contains a survey study with 109 parents. All the extracted data came from study 2, the interview study. The participants were selected from the participants of study 1.

SLT, speech and language therapist; SSD, speech sound disorder.

**TABLE 3c:** characteristics of included studies: Systematic reviews.

First author and year	Study aim	Study design and method	No. of papers	Further study details	Country
Sugden, 2016	To look at the available evidence detailing parental involvement in intervention studies for children with phonology‐based SSD	Systematic review	49	Peer‐reviewed, written in or translated into English, 1979–2013, evidence levels I (meta‐analysis)—III (case‐series)	Australia

*Note*: SSD, speech sound disorder.

### Review findings

**FIGURE 2:**
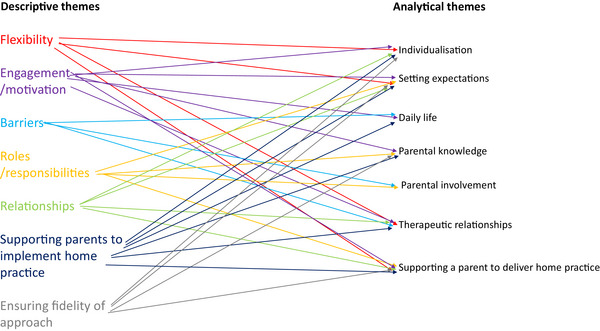
Development of the analytical themes from descriptive themes.

The team initially developed seven descriptive themes, and from these, seven broad analytical themes were developed with 16 underlying subthemes. A summary of themes and subthemes is presented in Table 4. Figure 2 demonstrates the links between the descriptive themes and the final analytical themes. The broad themes and subthemes spanned across the research questions.

**TABLE 4:** Themes and subthemes.

**Theme 1: Individualization**
Subtheme 1: Adapting to the parent/family
Subtheme 2: Adapting to the child
**Theme 2: Setting expectations**
Subtheme 1: Expectations of speech and language therapist (SLT)/parent role
Subtheme 2: Expectations of parental involvement within the session
Subtheme 3: Expectations about what to do at home
**Theme 3: Daily life**
Subtheme 1: Daily life barriers to home practice
Subtheme 2: Daily life facilitators
**Theme 4: Parental knowledge**
Subtheme 1: The importance of knowing why
Subtheme 2: The importance of knowing how
**Theme 5: Parental involvement**
Subtheme 1: Parental involvement is important to successful outcomes
Subtheme 2: Parental involvement is not always easy
**Theme 6: Therapeutic relationships**
Subtheme 1: Parent–child relationships
Subtheme 2: SLT–child relationships
Subtheme 3: SLT–parent relationships
**Theme 7: Supporting parents to deliver home practice**
Subtheme 1: Diversity of coaching methods
Subtheme 2: Monitoring

### Theme 1: Individualization

This theme explored how the SLT adapts an intervention approach to the family and child to support participation in home practice. This can be described in two subthemes: Adapting to the parent/family, and adapting to the child.

#### Adapting to the parent/family

Adapting the approach to the specific family and their context was found to be important to parents (Bear, 2022; McAllister et al., 2011; Sugden et al., 2019) and this was also recognized by SLTs (Furlong et al., 2018; Pappas et al., 2008; Sugden et al., 2018a; Tambyraja, 2020).

[H]ave a conversation with the parents about what they can manage at home. (Furlong et al., 2018: 1130)

Adaptations were related to different stages of the process. These included the target setting which was often done in consultation with the parents and took their views about what was important or manageable into consideration (Bear, 2022; Lefebvre et al., 2017). Home practice might also be adapted based on parental feedback or guided by the parent's/family's individual needs (Broen & Westman, 1990; Lim et al., 2020; Watts Pappas, 2010).

Although a structure for the training sessions was outlined, there was some flexibility with its delivery depending on the needs of the parent learner. (Lim et al., 2020, parent training sessions)

For approaches that aimed to provide a more naturalistic context for home practice, studies described how the parent has to adapt their approach to their own individual contexts with guidance from the SLT (Bowen & Cupples, 1999; Hartman et al., 1989; Lefebvre et al., 2017; Scherer et al., 2008).

[P]arents learn … to integrate these techniques into naturalistic contexts. (Bowen & Cupples, 1999: 42)

Feedback and praise can also be individualized to the parent and used to support their skill development (Eiserman et al., 1990; Flanagan & Ttofari Eecen, 2018; Furlong et al., 2018; Hartman et al., 1989; Scherer et al., 2008; Sugden et al., 2020; Tambyraja, 2020; Watts Pappas, 2010).

Observe the parent working with the child, after training, and provide feedback and encouragement. (Tambyraja, 2020: 1992)

Discussion with the parent was felt to be vital by parents and SLTs for successful individualization of intervention (Bear, 2022; Bowen & Cupples, 1999; Broen & Westman, 1990; Furlong et al., 2018; Hartman et al., 1989; Lefebvre et al., 2017; Rvachew & Brosseau‐Lapré, 2015; Sugden et al., 2016; Watts Pappas, 2010).

[H]ow important it is for the SLT to schedule time to discuss the content of the activities to make sure it is functional for the family. (Bear, 2022: 208)

#### Adapting to the child

As well as adapting to the parent or family more broadly, approaches are adapted to the child receiving the intervention. These adaptations may be to support a child's intrinsic motivation such as making resources linked to their specific interests and making it fun for them (Bowen & Cupples, 1999; Bowen et al., 2004; Eiserman et al., 1990; Lim et al., 2020; Sugden et al., 2020; Watts Pappas, 2010). Some approaches considered extrinsic motivation in the form of rewards which could be tailored to the child (Bowen et al., 2004; Günther & Hautvast, 2010; Lim et al., 2020).

The rewards were individual based on the interests and age of the child. (Günther & Hautvast, 2010: 349)

Some approaches considered adaptations to support a child's other needs or commitments and thus reduce the potential burden on them, an example of this is incorporating SLT tasks into other homework activities.

[S]everal of the children used daily reading tasks set by school as a context for speech practice. (Bear, 2022: 213)

### Theme 2: Setting expectations

This theme explored how SLTs set expectations during the intervention process and how parents understand these. This can be described by three subthemes: expectations of SLT/parent role, expectations of parental involvement within the session, and expectations of what to do at home.

#### Expectations of SLT/parent role

Some of the interventions explicitly included discussion of expectations about the parental and SLT roles (Bowen & Cupples, 1999; Lefebvre et al., 2017; Sugden et al., 2020; Watts Pappas, 2010). Some parents see the SLT as being in an expert role and changing this viewpoint is seen as important to SLTs. \

Some participants commented that educating parents about the role of an SLP^1^ was necessary to change a commonly held mindset that the SLP's role is to ‘fix their kid. (Furlong et al., 2018: 1130)

The view of SLTs in terms of their own role in the intervention process is less explored. There is some evidence that there is a conflict between SLTs’ expectations and beliefs about using a family centred approach and what they actually do in practice, which is often therapist led (Pappas et al., 2008).

#### Expectations of parental involvement within the session

Whilst all of the approaches that involve home practice have an expectation that the parent is involved in the direct therapy sessions, the nature of this expectation differs. Parents are not always expected to be involved in the whole session. However other approaches expect the parents to be involved in observation, demonstration, feedback and discussion throughout.

These procedures were demonstrated for the mothers. Each mother then practiced these procedures with her child in order for the therapist to provide feedback. (Eiserman et al., 1990: 301)

These expectations observed in the intervention studies do not appear to always translate into parents’ perceptions of their role in the direct therapy sessions.

When we go there … I let Susan do the work, it's not so much that's what she's paid for, it's not what I mean, but she's the speech pathologist, so she's to do the work. (Watts Pappas et al., 2016: 231)

#### Expectations about what to do at home

As well as having expectations during the direct therapy sessions, all of the intervention approaches had requirements for home practice that the parents were expected to follow through. Studies reported using written home programmes or homework books to support the parents with this.

The goals of therapy, the child's progress through treatment, and a description of the therapy, including homework material, were documented in a therapy journal. (Günther & Hautvast, 2010: 349)

As with the expectations of what to do within the direct therapy sessions, parents report that SLTs do not always communicate clearly to them about their role in home practice.

[P]arents also spoke about how their SLP had expectations about the parents’ role in home practice and that this was not always discussed. (Sugden et al., 2019: 170)

### Theme 3: Daily life

This theme explored the importance of home practice being incorporated into a child/family's daily life and factors that are barriers or facilitators to successful home practice.

#### Daily life barriers to home practice

Studies clearly reported that SLT home practice is not always easy to fit in. Families often have busy lives with competing commitments which make home practice difficult. Daily life barriers included things such as having other children, illness, holidays, and work commitments. SLTs perceive that family context can make engagement in home practice harder.

family involvement was further complicated by issues relating to social disadvantage, complex family dynamics and traumatic issues such as domestic violence, meaning that speech and language therapy was less of a priority. (Furlong et al., 2018: 1131)

#### Daily life facilitators

Conversely, when home practice considers a family's daily life and routine, this can be a facilitator and can be the key to success. Incorporating practice into the daily routine reduces the potential burden on families. From the perspective of parents:

Families reported that intervention needed to fit within their daily life and routines if they were to be able to engage in services. (McAllister et al., 2011: 262)

Fitting practice into a family's routine was also presented as a consideration for SLTs for many intervention approaches (Bear, 2022; Bowen & Cupples, 1999; Broen & Westman, 1990; Hartman et al., 1989; Lefebvre et al., 2017; Sugden et al., 2019; Watts Pappas, 2010).

Include practice activities that are part of the home routine so that homework doesn't seem time‐consuming or an extra task to try and fit in during the day. (Hartman et al., 1989: 6)

### Theme 4: Parental knowledge

This theme explored the importance of a parent's knowledge around the intervention; the theoretical rationale behind the approach and how to deliver it at home. The clarity with which information is delivered to a parent is an important component in parents delivering home practice with fidelity. It can be explored through two subthemes, the importance of knowing why and the importance of knowing how.

#### The importance of knowing why

Many of the intervention approaches support parents to understand why the therapy approach has been chosen by explaining the theory behind the approach (Bear, 2022; Bowen & Cupples, 1998; Eiserman et al., 1990; Günther & Hautvast, 2010; Lefebvre et al., 2017; Lim et al., 2020; Rvachew & Brosseau‐Lapré, 2015; Sugden et al., 2020).

Both the parent and the SLT voice indicated that a parent understanding why they are doing something is important and this supports parental engagement with the intervention process.

In order for families to engage in speech–language pathology services, they reported needing to be aware of what intervention would assist their child and how it would assist. (McAllister et al., 2011: 261)

#### The importance of knowing how

Not only is it important for parents to understand why they are doing something, they also need to understand how they need to do it in order for them to be successful. This is supported by ensuring that the parents have the time and space to ask for help or clarification about what they are doing (e.g., Ruscello et al., 1993). Being informed, about how to deliver intervention, using clear language was important to parents and played a role in building their confidence.

the importance of the SLT explaining things to parents in a way they can understand. (Watts Pappas et al., 2016: 233)

One of the ways that intervention approaches support parents to complete homework accurately and understand how to do it well is by only asking the parent to complete activities at home that they have been involved with or observed in the intervention session.

given homework tasks to do with the child that continued activities from the therapy session. (Lancaster et al., 2010: 178)

### Theme 5: Parental involvement

This theme highlighted the importance of parental involvement and explored how SLTs experience and perceive parental involvement as well as the barriers that are experienced by SLTs and parents. It can be broken into two subthemes: Parental involvement is important to successful outcomes, and parental involvement is not always easy.

#### Parental involvement is important to successful outcomes

SLTs believe that involving parents in intervention tasks at home is important to the success of an intervention and that without this, the child's speech will not improve (Furlong et al., 2018; McLeod & Baker, 2014; Pappas et al., 2008; Tambyraja, 2020).

The involvement of parents in doing homework is vital if any improvement is to occur. (Pappas et al., 2008: 340)

SLTs also believe that involving parents at home is an important factor to support them to deliver the intervention approach they choose, although this should not be relied on and may disadvantage some children (Furlong et al., 2018; Joffe & Pring, 2008; Sugden et al., 2018a).

Involving parents in therapy offers a potential solution to the lack of clinical time. … A concern here is that too great a reliance on parents might disadvantage those children whose parents are unwilling or unable to participate. (Joffe & Pring, 2008: 160)

#### Parental involvement is not always easy

Despite the perceived need to involve parents, both SLTs and parents report this to be difficult at times due to a variety of barriers. When SLTs were asked about barriers to parental involvement they predominantly discussed barriers related to the parents.

Most (61.8%) of the responses indicated that SLPs faced parent‐related barriers when attempting to train parents to provide intervention. (Sugden et al., 2018a: 773)

However, there was some discussion about the barriers that came from the SLT. These included a lack of skill and experience in working with parents of children with SSD and SLTs lack of time.

In response to the question ‘What is the reason for more or less parental involvement?’ … 11% of respondents reported therapist barriers including time constraints…and a lack of therapist confidence or experience in involving parents in intervention. (Pappas et al., 2008: 340)

Lack of time and lack of capability were also discussed with reference to parental barriers by both parents and SLTs.

[SLTs] associated poor compliance with the capabilities and confidence of parents to provide the therapy. (Furlong et al., 2018: 1131)

### Theme 6: Therapeutic relationships

This theme explored the therapeutic relationships and their role in the success of home practice. The evidence for this theme came from studies of intervention approaches or from parents. Whilst it is clear SLTs feel working with parents is vital, it is not documented how SLTs work to build relationships with parents and how important they feel different therapeutic relationships are. This theme is broken into three subthemes, parent–child relationship, SLT–child relationship, and the SLT–parent relationship.

#### Parent–child relationships

Working at home with their child can have a positive impact on a parent–child relationship and parents want to be involved and value being able to spend time with their child (Lim et al., 2020; Sugden et al., 2019; Watts Pappas et al., 2016).

All parents reported finding that the therapy sessions allowed them to spend some quality one‐on‐one time with their child. (Lim et al., 2020, positive relationship‐building)

This relationship in home practice can go beyond just the parent child relationship and can involve the whole family, which is perceived to be positive (Bear, 2022; Sugden et al., 2018a, 2019). When this support is not in place it can have a negative impact.

One mother spoke of:

the struggles of completing home practice when her extended family network was not involved or supportive. (Sugden et al., 2019: 171)

#### SLT–child relationship

A good relationship between the child and the SLT is seen as important to many of the approaches (Bear, 2022; Bowen & Cupples, 1999; Broen & Westman, 1990; Hartman et al., 1989; Ruscello et al., 1993; Rvachew & Brosseau‐Lapré, 2015; Sugden et al., 2020; Watts Pappas, 2010). Bowen's PACT approach (Bowen & Cupples, 1999), for example, ensures the SLT and child have one to one time to support this relationship. Parents feel that the SLT–child relationship is important for home practice, and when this relationship goes wrong it is detrimental to success and can result in families withdrawing from intervention.

[D]iscontinued private speech–language therapy after her child had a very negative experience with that SLT. (Watts Pappas et al., 2016: 232)

#### SLT–parent relationship

The relationship that the parent/family has with the SLT is also seen as important and can support parents to engage with the intervention. Parents may seek alternative support when they perceive their relationship with the SLT to be poor.

Some participants, such as Patrick's mother, reported not having a good relationship with the SLP and so having to go elsewhere. (McAllister et al., 2011: 262)

### Theme 7: Supporting parents to deliver home practice

This theme explored ways in which SLTs support parents to deliver home practice and the impact this can have on the fidelity of the approach as well as parents’ experiences of it. This is explored in two subthemes, diversity of coaching methods and monitoring.

#### Diversity of coaching methods

Throughout the intervention studies it is observed that a variety of methods are used to support the coaching of the parents (Bear, 2022; Bowen et al., 2004; Broen & Westman, 1990; Eiserman et al., 1990; Flanagan & Ttofari Eecen, 2018; Günther & Hautvast, 2010; Hartman et al., 1989; Lancaster et al., 2010; Lefebvre et al., 2017; Lim et al., 2020; Pumnum et al., 2015; Ruscello et al., 1993; Rvachew & Brosseau‐Lapré, 2015; Scherer et al., 2008; Sugden et al., 2020; Watts Pappas, 2010). These include a variety of written and verbal information as well as observation, feedback, use of scripts and role play. For the most part this diversity of approach to coaching is observed; however, in the most recent intervention study (Sugden et al., 2020) the diverse approach to coaching is explicitly discussed using the Dunst and Trivette (2009) model which includes: ‘(a) joint planning, (b) observation, (c) action, (d) reflection, and (e) feedback’ (Sugden et al., 2020: 116).

Parents reflected that learning occurred when a variety of approaches was used.

They (parents) referred to learning about the speech programme by having an explanation from the therapist, watching the videos, the therapist modelling the activities and the leaflet. (Bear, 2022: 199)

Practising SLTs also spoke of using a variety of means to help develop the parents’ skills and knowledge.

Participants ensured that the parents understood what was required for home practice by teaching and supporting them in a variety of ways. (Furlong et al., 2018: 1130)

#### Monitoring

The majority of approaches had some way of monitoring what the parents were doing at home with their child. This monitoring ranged from parents recording what or when they were practicing on a record sheet or book (Bowen & Cupples, 1998; Broen & Westman, 1990; Eiserman et al., 1990; Flanagan & Ttofari Eecen, 2018; Furlong et al., 2018; Günther & Hautvast, 2010; Hartman et al., 1989; Lim et al., 2020; Sugden et al., 2020) to parents being asked to audio or video record their home practice for the SLT to review (Bowen & Cupples, 1998; Eiserman et al., 1990; Furlong et al., 2018; Lim et al., 2020; Tambyraja, 2020).

The parents kept a diary of homework practice to track the number of practice sessions per week, number of trials per practice session and accuracy of the child's productions. (Flanagan & Ttofari Eecen, 2018: 213)

However, engagement with home practice activities was not always followed up with parents in subsequent SLT sessions.

The rate of reported follow‐up by SLPs to parents about completion of homework activities was considerably lower than that of initially sending homework out. (Tambyraja, 2020: 1993)

## DISCUSSION

This review set out to explore the perceptions, experiences and strategies underpinning effective collaborative working between SLTs and parents of children, aged ≤ 5;11 with SSD. The key findings in relation to the review questions were as follows:

1. That SLTs use a range of coaching methods, individualizing their approach to suit the family, allowing them to fit practice into their daily life.
2. That delivering information to parents with clarity and monitoring the delivery of the intervention through use of observation or video can support parents to deliver home practice with fidelity.
3. That SLTs believe that working with parents is vital for children with SSD to make progress in their intervention but that this is not always easy due to a multitude of barriers.
4. That positive therapeutic relationships between SLTs, parents and their children motivate parents to take part in home practice. Parents value intervention that is individualized to their family context, fitting in home practice is not always easy.

The PAGER framework (Bradbury‐Jones et al., 2022) was used to inform the discussion of the core patterns (or analytical themes) arising from this scoping review in light of advances in practice, gaps in practice and recommendations for future research, and evidence for practice and (Table 5).

**TABLE 5:** PAGER framework and mapping to research questions.

Patterns (analytical themes)	Relevant research questions	Advances	Gaps	Evidence for practice	Research recommendations
1. Individualization	1, 3, 4	There is growing evidence from parents that an individualized approach is valued by parents and makes their experience easier. Approaches can be individualized to the child and/or the family. How to adapt the approaches to the individual was a consideration in most of the intervention studies	How flexible is too flexible and what elements of an approach allow for this flexibility whilst maintain fidelity and which ones do not? Is it possible for a parent to individualize home practice whilst maintaining fidelity and how much guidance does a parent need to do this?	Work with parents and children to make sure the targets set, and home practice activities are meaningful to them	Comparative intervention studies looking at flexible approaches versus prescribed approaches
2. Setting expectations	1, 2, 4	Many of the approaches included expectations around intervention intensity and the monitoring of the home practice. Parents value when expectations about their role and what they need to do are clear, this is not always their experience	There is limited knowledge about how SLTs set expectations with parents and what is the best way to do so to ensure they are clear	Develop a shared understanding with parents about the SLT and parent role within the intervention process. Parents value the SLT agreeing clear expectations around home practice	Research with SLTs and parents to find out the best way to set up expectations so that they are clear for both parties
3. Daily life	1, 3, 4	Flexibility to incorporate home practice into a family's daily life can facilitate parents to work at home with their child. However, daily life can also create barriers to home practice	It is not known what factors make fitting home practice into family life easy or difficult for parents and why some parents manage to complete it despite busy lives and others do not	Working with parents to determine what is possible at home and providing clear expectations for home practice, will support the fidelity of intervention by ensuring evidence‐based intensity is able to be met within the confines of the family's daily life	Research that continues to explore the barriers and facilitators for successful home practice
4. Parental knowledge	2–4	Intervention approaches increasingly include discussion and education around the rationale for the approach, including parents in the informed decision making. Parents value knowing why and how they are doing something, and this supports their engagement in the intervention process	There is a lack of specificity in the literature about what SLTs and parents think work in developing parents’ knowledge, as well as approaches that measure parental knowledge development as a specified variable	Parental coaching needs to ensure that parents understand the how and why of the intervention	Research to draw on lived experiences of what has worked to support the development of parents’ knowledge AND intervention studies that measure the parents’ acquisition of skills and knowledge and the impact this has on the outcomes of intervention
5. Parental involvement	3, 4	SLTs believe that working with parents is vital for the success of their intervention. SLTs and parents feel that this is not always easy	Whilst SLTs believe working with parents is important there is some evidence that SLTs do not have the training, confidence or knowledge to know how to support parents to work at home	Work with parents and children to make sure the targets set, and home practice activities are meaningful to them	Development of the SLT graduate and postgraduate training, drawing on behaviour change theory, models of adult learning and participatory research with parents and SLTs. This should support SLTs with coaching parents to understand and develop their role as implementors of intervention, building therapeutic relationships, setting expectations and how to engage diverse groups of parents in the process
6. Therapeutic relationships	2, 4	Relationships between SLT/parent, child/SLT and parent/child are all important to the success of an intervention. Parents value all of these relationships and without these may disengage from intervention	SLTs perspectives of the impact of therapeutic relationships is largely undocumented, aside from the belief that working together with parents is important	Spending time fostering positive therapeutic relationships between SLTs, the parent and the child to ensure everyone is working together is valued by parents and supports effective, evidence‐based intervention	Research with SLTs to explore their perspectives and experiences of therapeutic relationships and the importance of these to outcomes
7. Supporting parents to deliver home practice	1–4	Using a variety of methods supports parents to deliver home practice. More recent approaches consider this in relation to theories of adult learning and include parental reflection as part of the coaching	There is a current lack of specificity in the literature about what parents or practising SLTs feel are the important active ingredients for intervention at home to be successful	Use a diverse range of coaching techniques to support a variety of learning styles. This will support the development of parents’ skills and knowledge	Research with SLTs and parents to explore their experiences of what makes the support a parent provides at home successful

### Advances

Due to the wide date range of the sources included, this review highlights that there have been significant changes to the perception of the role of the parent and SLT within the therapeutic process for children with SSD. Working with parents to support children with SSD was historically discussed in intervention manuals (Hartman et al., 1989) before going on to be included in research studies, and is now seen as vital to both parents and SLTs. Until 10 years ago studies focussing on working with parents were few and sporadic; however, research in this area has accelerated recently (Bear, 2022; Flanagan & Ttofari Eecen, 2018; Furlong et al., 2018; Lefebvre et al., 2017; Lim et al., 2020; McLeod & Baker, 2014; Pumnum et al., 2015; Rvachew & Brosseau‐Lapré, 2015; Sugden et al., 2016, 2018a, 2020, 2019; Tambyraja, 2020; Watts Pappas et al., 2016).

A diverse range of approaches to coaching parents has been used in many studies and this diversity is supported by theoretical approaches to adult learning theories (e.g., Dunst & Trivette, 2009, 2012) but it is only more recently that the link has been explicitly made by researchers (Sugden et al., 2020). This explicit link has also seen the inclusion of parental reflection as part of the intervention which is considered key in such models for retention of knowledge. This relationship between adult learning theory and how SLTs engage with parents has been explored more explicitly in other areas of SLT (Bellon‐Harn et al., 2020; Klatte et al., 2020) but is relatively unexplored in the field of SSD. There has also been an increased body of literature over the last 20 years looking at how parents and SLTs experience and perceive working together, something relatively unexplored, until recently for children with SSD (Furlong et al., 2018; Joffe & Pring, 2008; McAllister et al., 2011; McLeod & Baker, 2014; Sugden et al., 2018a, 2019; Tambyraja, 2020; Watts‐Pappas et al., 2016).

### Gaps and recommendations for research

Individualization, including adapting to the family's daily activities has been found to be important but there is a lack of information about how flexible is too flexible and when flexibility to support home practice impacts on the fidelity of the approach, and efficacy. Intervention studies that compare flexible, individualized approaches to prescribed approaches would support SLTs to know how flexible they can be with their approach to home practice.

There continues to be limited knowledge of how SLTs experience and perceive working with parents and how SLTs develop relationships with parents of children with SSD and the importance of these on outcomes. There is also a lack of specificity about what SLTs and parents feel are the most effective ingredients to delivering home practice successfully. Further research with parents and SLTs will help to explore these areas in more depth.

Despite there being an expectation, within UK clinical guidelines (RCSLT, 2018), that SLTs should work with parents of children with SSD, information from SLTs suggests that there is a gap in SLTs’ skills and knowledge impacting their ability and confidence when working with parents (Pappas et al., 2008; Sugden et al., 2018a). This indicates the potential need to develop the SLT pre‐registration curriculum to include teaching around working with and coaching parents more explicitly. Development and trialling of a specific training package for SLTs working with parents of children with SSD may support the development of already qualified SLTs skills and knowledge in this area. Research into the application of behaviour change theories (e.g., Michie et al., 2011, behaviour change wheel) in the field of SLT (e.g., Baker et al., 2024; Barnett et al., 2023) has gathered recent interest and may be important to the development of such teaching and training programmes which will need to consider how the SLT themselves change the way in which they work with parents, as well as supporting the parents to change their behaviour to include regular home practice with their child. The impact that this additional training has on clinical practice could then be evaluated.

The results of this review also highlight how supporting parents in multiple ways, with flexibility, whilst taking time to build relationships can be a powerful way to overcome some of the barriers faced by parents. However, it should be noted that the literature suggests that some barriers are still currently insurmountable and further research is needed in order for SLTs to support all parents effectively. Such research may support SLTs to reduce the potential inequalities within their service and thus provide an equitable offer.

### Evidence for practice

Working jointly with parents in intervention to support successful home practice has the potential to improve the success of intervention for children with SSD. However, successful home practice relies on a number of factors. The following recommendations could be considered by SLTs working with young children with SSD and their families:

- Spending time fostering positive therapeutic relationships between SLTs, the parent and the child to ensure everyone is working together is valued by parents and supports effective, evidence‐based intervention.
- Work with parents to determine what is possible at home and provide clear expectations for home practice to support the fidelity of intervention by ensuring evidence‐based intensity can be delivered within the confines of the family's daily life.
- Work with parents and children to make sure the targets set and home practice activities are meaningful to them.
- Develop a shared understanding with parents about the SLT and parent role within the intervention process.
- Use a diverse range of coaching techniques to support a variety of learning styles. This will support the development of parents’ skills and knowledge.
- Parental coaching needs to ensure that parents understand the how and why of the intervention.

### Limitations of evidence

The parent participants in the studies were mostly mothers. There is limited evidence in the current literature about how fathers or other caregivers experience and perceive SLT for their children with SSD. It should not be assumed that the findings from this synthesis can be used by SLTs when working with fathers or other caregivers who may be involved in a child's SLT. The majority of studies were from two English‐speaking countries (Australia and the United States). This was influenced by the scope of the study not allowing for the translation of papers into English from other languages. Four sources were excluded purely on the basis that they were not published in English, and of these, three were from Europe. However, it is possible that this is a limitation of the evidence itself, demonstrating that there is a lack of evidence found from the Global South in this field. The implications of this in terms of the impact of cultural differences and expectations of how SLTs and parents work together and how this is perceived in different parts of the world is not addressed in the current research for children with SSD.

## CONCLUSIONS

Despite these limitations, emerging evidence supports the value of SLTs and parents working together to support home practice for children with SSD. The findings of this study highlight existing knowledge which will support SLTs to work optimally with parents to implement home practice for their child with SSD. Taking time to foster working relationships with parents supports effective home practice and SLTs need to take a flexible approach to this. The review identifies gaps in the current skills and knowledge of SLTs, highlighting the need for further research, support and guidance for SLTs in their work with parents, as well as implications for the development of the SLT pre‐registration curriculum.

## CONFLICT OF INTEREST STATEMENT

Dr Jill Titterington is Editor‐in Chief of the *IJLCD*.

## Supporting information



Supporting Information

Supporting Information

Supporting Information

## Data Availability

The data that support the findings of this study are available in the supplementary material of this article.

## ENDNOTE

1 SLP stands for speech and language pathologist and is a common term for speech and language therapist in Australia where this research was conducted.
