# Supplementary material for: How speech and language therapists and parents work together in the therapeutic process for children with speech sound disorder: A scoping review
Source: Int J Lang Commun Disord. 2024 Nov 18;60(1):e13132. doi: 10.1111/1460-6984.13132 (PMC11606383; doi:10.1111/1460-6984.13132)
Supplement: Supplementary file 1 — Supporting Information [file JLCD-60-0-s003.docx]

## Appendix 1 – full search strategy for scoping review

| Date | Database | Search |
| --- | --- | --- |
| 07.02.23 | pubmed | (("Speech Sound disorder*"OR “phonological disorder*"OR "articulation disorder*"OR "motor speech disorder*" OR "consistent phonological disorder*" OR "phonological delay" OR "inconsistent phonological disorder*" OR "speech difficult*" OR "speech disorder*" OR "speech delay" OR "speech development" OR "speech intelligibility") AND (intervention* OR coaching OR support OR treatment*)) AND ("parent training" OR "parental training" OR homework OR "home programme" OR "parent involvement" OR "parental involvement" OR "indirect" OR "parent implemented" OR "parent led" OR "parent based" OR "parent engagement" OR "parental engagement" OR "family engagement" OR "parent participation" OR "professional-family relations*" OR "family participation" OR "family implemented" OR "family training") MESH |
| 07.02.23 | psychinfo | (("Speech Sound disorder*"OR "phonological disorder*"OR "articulation disorder*"OR "motor speech disorder*" OR "consistent phonological disorder*" OR "phonological delay" OR "inconsistent phonological disorder*" OR "speech difficult*" OR "speech disorder*" OR "speech delay" OR "speech development" OR "speech intelligibility") AND (intervention* OR coaching OR support OR treatment*)) AND ("parent training" OR "parental training" OR homework OR "home programme" OR "parent involvement" OR "parental involvement" OR "indirect" OR "parent implemented" OR "parent led" OR "parent based" OR "parent engagement" OR "parental engagement" OR "family engagement" OR "parent participation" OR "professional-family relations*" OR "family participation" OR "family implemented" OR "family training")  (Map to subject headings) |
| 07.02.23 | EBSCOhost  (including ERIC, ERC, BEI, CDAS) | (("Speech Sound disorder*" OR "phonological disorder*"OR "articulation disorder*" OR "motor speech disorder*" OR "consistent phonological disorder*" OR "phonological delay" OR "inconsistent phonological disorder*" OR "speech difficult*" OR "speech disorder*" OR "speech delay" OR "speech development" OR "speech intelligibility") AND (intervention* OR coaching OR support OR treatment*)) AND ("parent* training" OR homework OR "home programme" OR "parent* involvement" OR "indirect" OR "parent implemented" OR "parent led" OR "parent based" OR "parent* engagement" OR "family engagement" OR "parent* participation" OR "professional-family relations*" OR "family participation" OR "family implemented" OR "family training") |
| 09.02.23 | web of science core collection | (("Speech Sound disorder*"OR "phonological disorder*"OR "articulation disorder*"OR "motor speech disorder*" OR "consistent phonological disorder*" OR "phonological delay" OR "inconsistent phonological disorder*" OR "speech difficult*" OR "speech disorder*" OR "speech delay" OR "speech development" OR "speech intelligibility") AND (intervention* OR coaching OR support OR treatment*)) AND ("parent training" OR "parental training" OR homework OR "home programme" OR "parent involvement" OR "parental involvement" OR "indirect" OR "parent implemented" OR "parent led" OR "parent based" OR "parent engagement" OR "parental engagement" OR "family engagement" OR "parent participation" OR "professional-family relations*" OR "family participation" OR "family implemented" OR "family training") |
| 09.02.23 | Scopus | (("Speech Sound disorder*"OR "phonological disorder*"OR "articulation disorder*"OR "motor speech disorder*" OR "consistent phonological disorder*" OR "phonological delay" OR "inconsistent phonological disorder*" OR "speech difficult*" OR "speech disorder*" OR "speech delay" OR "speech development" OR "speech intelligibility") AND (intervention* OR coaching OR support OR treatment*)) AND ("parent training" OR "parental training" OR homework OR "home programme" OR "parent involvement" OR "parental involvement" OR "indirect" OR "parent implemented" OR "parent led" OR "parent based" OR "parent engagement" OR "parental engagement" OR "family engagement" OR "parent participation" OR "professional-family relations*" OR "family participation" OR "family implemented" OR "family training") |
| 10.02.23 | Cinahl | (("Speech Sound disorder*"OR "phonological disorder*"OR "articulation disorder*"OR" motor speech disorder*" OR "consistent phonological disorder*" OR "phonological delay" OR "inconsistent phonological disorder*" OR "speech difficult*" OR "speech disorder*" OR "speech delay" OR "speech development" OR "speech intelligibility") AND (intervention* OR coaching OR support OR treatment*)) AND ("parent training" OR "parental training" OR homework OR "home programme" OR "parent involvement" OR "parental involvement" OR "indirect" OR "parent implemented" OR "parent led" OR "parent based" OR "parent engagement" OR "parental engagement" OR "family engagement" OR "parent participation" OR "professional-family relations*" OR "family participation" OR "family implemented" OR "family training") |
| 10.02.23 | Cochrane | ("Speech Sound disorder") OR "phonological disorder" OR "articulation disorder" OR "motor speech disorder" OR "consistent phonological disorder" OR "phonological delay" OR "inconsistent phonological disorder*" OR "speech difficult*" OR "speech disorder" OR "speech delay" OR "speech development" OR "speech intelligibility") AND (intervention* OR coaching OR support OR treatment*) AND ("parent training" OR "parental training" OR homework OR "home programme" OR "parent involvement" OR "parental involvement" OR "indirect" OR "parent implemented" OR "parent led" OR "parent based" OR "parent engagement" OR "parental engagement" OR "family engagement" OR "parent participation" OR "professional-family relations" OR "family participation" OR "family implemented" OR "family training") |
| 10.02.23 | LLBA | (("Speech Sound disorder*"OR "phonological disorder*"OR "articulation disorder*"OR "motor speech disorder*" OR "consistent phonological disorder*" OR "phonological delay" OR "inconsistent phonological disorder*" OR "speech difficult*" OR "speech disorder*" OR "speech delay" OR "speech development" OR "speech intelligibility") AND (intervention* OR coaching OR support OR treatment*)) AND ("parent training" OR "parental training" OR homework OR "home programme" OR "parent involvement" OR "parental involvement" OR "indirect" OR "parent implemented" OR "parent led" OR "parent based" OR "parent engagement" OR "parental engagement" OR "family engagement" OR "parent participation" OR "professional-family relations*" OR "family participation" OR "family implemented" OR "family training")  (Anywhere but full text) |
| 10.02.23 | proquest | (("Speech Sound disorder*"OR "phonological disorder*"OR "articulation disorder*"OR "motor speech disorder*" OR "consistent phonological disorder*" OR "phonological delay" OR "inconsistent phonological disorder*" OR "speech difficult*" OR "speech disorder*" OR "speech delay" OR "speech development" OR "speech intelligibility") AND (intervention* OR coaching OR support OR treatment*)) AND ("parent training" OR "parental training" OR homework OR "home programme" OR "parent involvement" OR "parental involvement" OR "indirect" OR "parent implemented" OR "parent led" OR "parent based" OR "parent engagement" OR "parental engagement" OR "family engagement" OR "parent participation" OR "professional-family relations*" OR "family participation" OR "family implemented" OR "family training")  (Anywhere but full text) |
