# Supplementary material for: How speech and language therapists and parents work together in the therapeutic process for children with speech sound disorder: A scoping review
Source: Int J Lang Commun Disord. 2024 Nov 18;60(1):e13132. doi: 10.1111/1460-6984.13132 (PMC11606383; doi:10.1111/1460-6984.13132)
Supplement: Supplementary file 2 — Supporting Information [file JLCD-60-0-s002.docx]

## Appendix 2 – data extraction forms and instructions for scoping review

#### For surveys, interviews and focus group studies

| **DATA EXTRACTION**  **Evidence source details and characteristics** | |
| --- | --- |
| Citation details (reference) |  |
| Type of study |  |
| Country |  |
| **Methodology and participants details including:**   - How participants were selected - Inclusion/exclusion criteria - Number of participants - Settings/client group work in/with (if SLTs) - Any other details (e.g., % of SSD specialists) - Languages spoken | If possible, please capture the details listed, they may not all be available in every paper. Other things that may be of note could be years of experience. |
| Level of reported parental involvement/number of SLTs who involved parents | Anything that reports the level of parental involvement or how many SLTs involve parents etc It may not be explicitly reported so if not just leave blank |
| Aims | Specific research questions or overall aim of source |
| **Extracted content** | |
| **Q1.** How do SLTs support parents to develop their role as implementers of intervention in clinical sessions, alongside the SLT and in home-based activities, with children diagnosed with SSD up to age 5;11? | In here please extract any reports of how the SLT has involved parents, this could be reports of using written information, parental education groups, whether they included homework and the nature of this, observing sessions, discussion, peer support, using technology etc. |
| **Q2.** How do SLTs work with parents to ensure that the approach and intensity of intervention are delivered with fidelity in the home environment? | In here please extract anything that suggests there is some quality assurance to the parental involvement or ways in which SLTs are supporting parents to complete homework. This could be things like SLT observing parent with child and providing feedback, tailoring homework to child/family situation, use of homework diary, monitoring of homework and/or of parental skills, setting clear expectations around things like amount of home practice (e.g. studies that specify to the parents they must do 5mins a day etc), use of video recordings, parental reflection with SLT etc |
| **Q3.** How do SLTs experience and perceive working with parents? | This could be things such as perceived importance of the involvement, reported barriers or facilitators to involvement, things SLTs think are important to include in their work with parents. |
| **Q4.** How do parents experience and perceive working with therapists as implementers of intervention? | This could be anything that parents have reported about working alongside the therapist. Things like what makes it easier or more difficult, about the relationship with the SLT, barriers and facilitators from their perspective etc. |

#### For intervention studies

| **DATA EXTRACTION**  **Evidence source details and characteristics** | |
| --- | --- |
| Citation details (reference) |  |
| Type of study |  |
| Country |  |
| Nature of intervention | Just the type of intervention – this may be a specific technique (e.g., multiple oppositions) or could be eclectic/mix of approaches. If a mix, then please specify whether it is a mixed but purely phonological/phonetic/motor based approach or a mix of these. |
| Outcome of intervention |  |
| **DELIVERY:**   - Setting (e.g., home, clinic, nursery) - Deliverer (e.g., SLT consultative with parent, SLT only, if education setting is involved) - How working with the parent is included - Dosage - Duration |  |
| Participant details, including:   - Number of - Age range as reported - Diagnosis – including those excluded - Mono/multi-lingual - Language/s spoken |  |
| Aims |  |
