# Supplementary material for: How speech and language therapists and parents work together in the therapeutic process for children with speech sound disorder: A scoping review
Source: Int J Lang Commun Disord. 2024 Nov 18;60(1):e13132. doi: 10.1111/1460-6984.13132 (PMC11606383; doi:10.1111/1460-6984.13132)
Supplement: Supplementary file 3 — Supporting Information [file JLCD-60-0-s001.docx]

## Appendix 3 – instructions from first author to support analytical theme development prior to agreeing these as a team

**Process for developing analytical themes (analysing the results in relation to the questions)**

**Questions**

1. How do SLTs support parents to develop their role as implementers of intervention in clinical sessions, alongside the SLT and in home-based activities, with children diagnosed with SSD up to age 5;11?
2. How do SLTs work with parents to ensure that the approach and intensity of intervention are delivered with fidelity in the home environment?
3. How do SLTs experience and perceive working with parents?
4. How do parents experience and perceive working with therapists as implementers of intervention?

**Process**

- Look at each descriptive theme separately and make links between these and the questions. Noting what comes out from the theme in relation to the question.

For example:


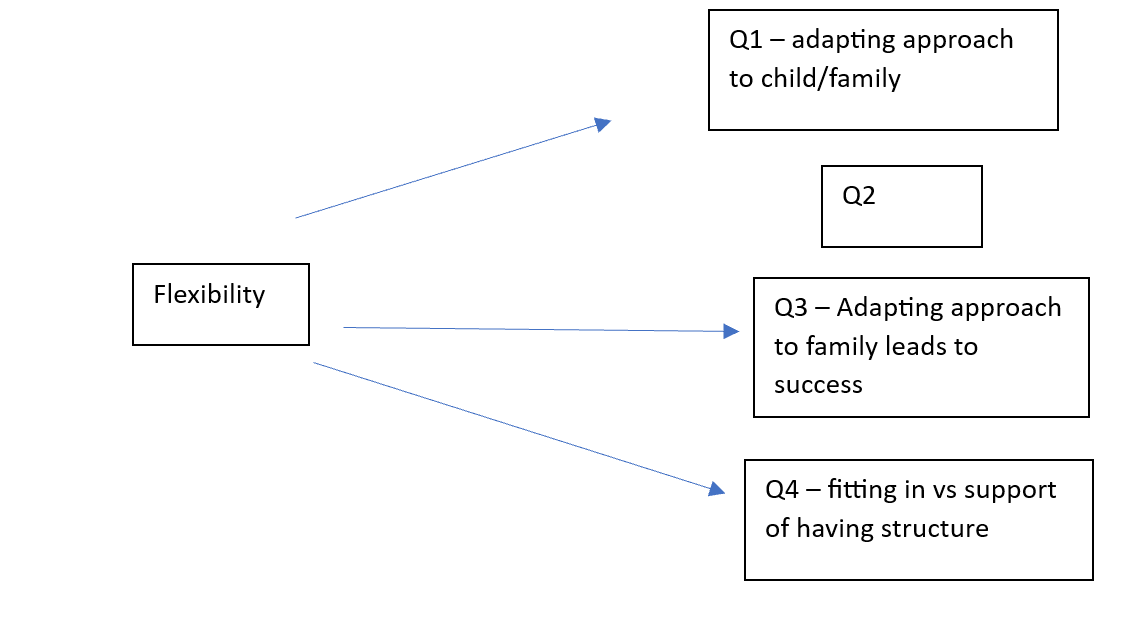


- Next take each question and the themes with the questions. For example:


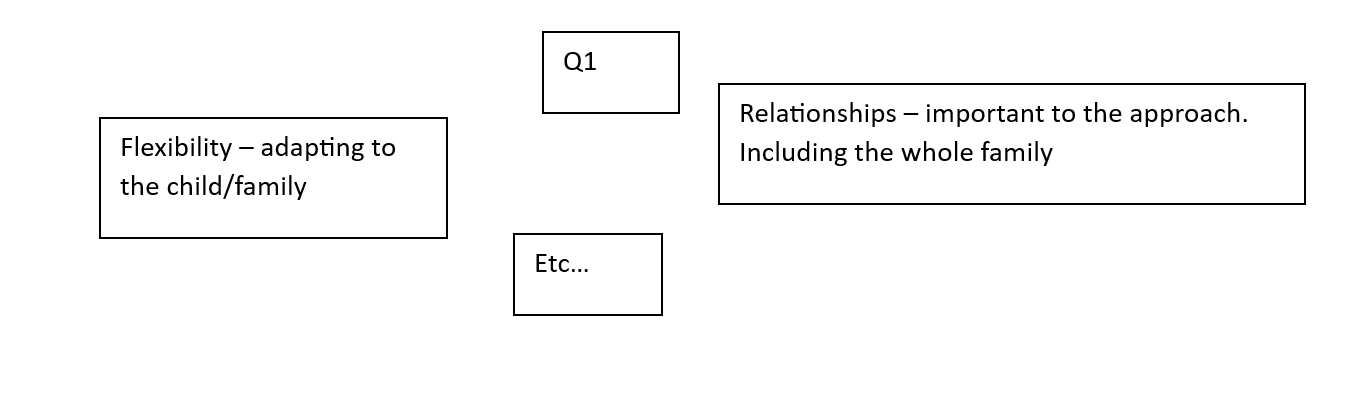


- Look as a whole and look for links across the question to start to come up with initial analytical themes. These should be interpretations of the information rather than a description of the data.
- Write a short summary about each analytical theme to ensure they are justifiable and then refine the themes accordingly to come up with potential analytical themes for each question (between 1-3 for each question) ready to discuss as a team.
